# Corticofugal GABAergic projection neurons in the mouse frontal cortex

**DOI:** 10.3389/fnana.2015.00133

**Published:** 2015-10-28

**Authors:** Ryohei Tomioka, Kenji Sakimura, Yuchio Yanagawa

**Affiliations:** ^1^Department of Morphological Neural Science, Graduate School of Medical Sciences, Kumamoto UniversityKumamoto, Japan; ^2^Department of Cellular Neurobiology, Brain Research Institute, Niigata UniversityNiigata, Japan; ^3^Department of Genetic and Behavioral Neuroscience, Gunma University Graduate School of MedicineMaebashi, Japan

**Keywords:** corticofugal pathways, GABAergic neurons, neocortex, mouse, somatostatin

## Abstract

Cortical projection neurons are classified by hodology in corticocortical, commissural and corticofugal subtypes. Although cortical projection neurons had been regarded as only glutamatergic neurons, recently corticocortical GABAergic projection neurons has been also reported in several species. Here, we demonstrate corticofugal GABAergic projection neurons in the mouse frontal cortex. We employed viral-vector-mediated anterograde tracing, classical retrograde tracing, and immunohistochemistry to characterize neocortical GABAergic projection neurons. Injections of the Cre-dependent adeno-associated virus into glutamate decarboxylase 67 (GAD67)-Cre knock-in mice revealed neocortical GABAergic projections widely to the forebrain, including the cerebral cortices, caudate putamen (CPu), ventral pallidum (VP), lateral globus pallidus (LGP), nucleus accumbens, and olfactory tubercle (Tu). Minor GABAergic projections were also found in the mediodorsal thalamic nucleus, diagonal band of Broca, medial globus pallidus, substantial nigra, and dorsal raphe nucleus. Retrograde tracing studies also demonstrated corticofugal GABAergic projection neurons in the mouse frontal cortex. Further immunohistochemical screening with neurochemical markers revealed the majority of corticostriatal GABAergic projection neurons were positive for somatostatin (SS)-immunoreactivity. In contrast, corticothalamic GABAergic projection neurons were not identified by representative neurochemical markers for GABAergic neurons. These findings suggest that corticofugal GABAergic projection neurons are heterogeneous in terms of their neurochemical properties and target nuclei, and provide axonal innervations mainly to the nuclei in the basal ganglia.

## Introduction

The overwhelming majority of long-range corticocortical connections in the cerebral cortex originate from glutamatergic pyramidal neurons. Recently, several investigations in rodents, carnivores, and monkeys have provided evidence that a small number of inhibitory nonpyramidal neurons have long-range corticocortical connections (Peters et al., 1990; McDonald and Burkhalter, 1993; Tomioka et al., 2005; Tomioka and Rockland, 2007; Higo et al., 2007, 2009). Although knowledge about corticocortical GABAergic projection neurons still remains sparse, growing evidence suggests that the vast majority of corticocortical GABAergic projection neurons are classified as the somatostatin (SS)-expressing neurons (Tomioka et al., 2005; Tomioka and Rockland, 2007; Higo et al., 2007, 2009; Jinno et al., 2007; McDonald et al., 2012; Melzer et al., 2012; McDonald and Zaric, 2015a,b). This knowledge about corticocortical GABAergic projection neurons has been most essential to estimate their functional significance until now (Tamamaki and Tomioka, 2010), since each GABAergic subpopulation classified by neurochemical properties would have the same morphological, and electrophysiological property (Markram et al., 2004; Ascoli et al., 2008).

Although corticofugal projection neurons had been considered as glutamatergic pyramidal neurons, two groups have recently shown that a subset of corticofugal afferents originates from GABAergic neurons (Jinno and Kosaka, 2004; Lee et al., 2014). Jinno and Kosaka (2004) have demonstrated that approximately 5% of corticostriatal neurons are parvalbumin (PV)-expressing GABAergic neurons in the mouse somatosensory cortex. Lee et al. (2014) have also found that GABAergic axonal fibers from the medial frontal cortex widely distribute in various subcortical regions: the caudate putamen (CPu), nucleus accumbens, claustrum, and basolateral amygdala. Besides, they have identified corticofugal GABAergic projection neurons as PV- or vasoactive intestinal peptide (VIP)-expressing subpopulations of GABAergic neurons. Those reports indicate corticofugal GABAergic projection neurons may be a distinct subpopulation from corticocortical GABAergic projection neurons because of their different neurochemical properties.

In this study, in order to examine the anatomical and neurochemical details of GABAergic projection neurons, we employed viral-vector-mediated anterograde tracing with glutamate decarboxylase 67 (GAD67)-Cre knock-in mice, classical retrograde tracing in GAD67-green fluorescent protein (GFP) knock-in mice. Improved detection of GABAergic neurons could provide more in depth characterization of GABAergic projections neurons in the mouse frontal cortex.

## Materials and Methods

### Animals

All experiments were approved by the Committee for Animal Experiments of Kumamoto University, and were performed in accordance with the Guidelines for Use of Animals in Experiments of Kumamoto University. All efforts were made to minimize animal suffering and the number of animals used. Two lines of transgenic mice targeting GABAergic neurons via GAD67 promoter were used in this study: GAD67-Cre knock-in mice (Higo et al., 2009) and GAD67-GFP knock-in mice (Tamamaki et al., 2003).

### Producing Anti-mCherry Antibody

We carried out the production of affinity purified anti-mCherry antibody, as we have previously described in Tomioka and Rockland (2006). Briefly, the cDNA fragment encoding the full-length of mCherry was subcloned into the pGEX-4T2 vector (GE Healthcare; Piscataway, NJ, USA) for expression of glutathione S-transferase (GST) fusion protein. GST-mCherry fusion protein was induced in Escherichia coli by adding isopropyl-1-thio-beta-D-galacto-pyranoside to the medium. GST-free mCherry were prepared by in-column thrombin digestion of GST fusion proteins bound to glutathione–Sepharose 4B media, according to the protocol recommended by the manufacturer of the GST system (GE Healthcare). Purified mCherry was emulsified with complete Freund’s adjuvant (Difco; Detroit, MI, USA) and injected intracutaneously into two female rabbits (1 mg/animal). Three weeks after the first immunization, the same amount of mCherry emulsified with complete Freund’s adjuvant was reinjected into the rabbits. Additional immunizations were performed every 2 weeks thereafter. After the third immunization, serum was taken from the rabbits. Antibodies were affinity purified with mCherry-conjugated Affigel 10 gel (2 mg mCherry/1 ml gel; BioRad, Richmond, CA, USA). The rabbit serum (1 ml) was applied to 1 ml of the antigen column, and the specific antibodies were eluted with 0.1 M glycine-HCl (pH 2.5) and mixed with 1 M potassium phosphate buffer (10:1) to achieve neutral pH. Antibody was stored at 4°C with 0.02% NaN3.

### Anterograde Labeling of Neocortical GABAergic Neurons Using the Adeno Associated Viruses

Two recombinant adeno-associated viruses serotype 5 (AAVs) were used in this study. One of AAVs, AAV-Ef1a-DIO-hChR2-mCherry, drives Cre-dependent channel rhodopsin-fluorescent protein fusion (Sohal et al., 2009), and another AAV-CAG-GFP drives GFP in various cells such as pyramidal, nonpyramidal, and glia cells. The GAD67-Cre knock-in mice (8–12 weeks) were anesthetized by intraperitoneal injection of chloral hydrate (30 mg/100 g body weight) and placed in a stereotaxic apparatus. To visualize only GABAergic neurons, 50 nl of AAV-Ef1a-DIO-hChR2-mCherry (1.6 × 10e12) in 110 mM NaCl and 1.56% sorbitol was injected into the frontal cortex of GAD67-Cre knock-in mice (2.3 mm anterior to the bregma, 1.5 mm lateral to the midline, and 1.5 mm deep from the brain surface) by pressure through a glass micropipette. In some experiments, to visualize both glutamatergic and GABAergic neurons, 30 nl of a mixture of AAV-CAG-GFP (1.8 × 10e11/ml) and AAV-Ef1a-DIO-hChR2-mCherry (1.4 × 10e12/ml) in 110 mM NaCl and 1.56% sorbitol was injected into the frontal cortex of a GAD67-Cre knock-in mice. The virus-injected mice survived for 3–4 weeks after the injection.

### Retrograde Labeling of Neocortical GABAergic Neurons Using Fast Blue

The GAD67-GFP knock-in mice (8–12 weeks) were anesthetized by intraperitoneal injection of chloral hydrate and placed in a stereotaxic apparatus. Less than 100 nl of 1% Fast Blue solution (FB; Sigma, St. Louis, MO, USA) dissolved in distilled water was injected into the ventral part of CPu (0.0 mm anterior to the bregma, 2.0 mm lateral to the midline, and 4.0 mm deep from the brain surface), mediodorsal thalamus (1.8 mm posterior to the bregma, 0.5 mm lateral to the midline, and 3.2 mm deep from the brain surface), or dorsal raphe nucleus (4.7 mm posterior to the bregma, 0.5 mm lateral to the midline, and 2.0 mm deep from the brain surface) by pressure through a glass micropipette. The FB-injected mice survived for 5–7 days after the injection.

### Fixation

The mice were deeply anesthetized by intraperitoneal injection of chloral hydrate (100 mg/100 g body weight), and perfused transcardially with 5 ml of PBS [0.9% (w/v) saline buffered with 5 mM sodium phosphate, pH 7.4], followed by 40 ml of PBS containing 4% (w/v) formaldehyde. After the mice were left for 2 h, the brains were removed and immersed in 30% sucrose in PBS overnight for cryoprotection. The brains were cut into 40 μm-thick frontal sections on a freezing microtome.

### Immunoperoxidase Staining for Anterograde Labeling

In order to enhance the immunoreactivity of anterograde labeling, we employed the method of combining the avidin biotinylated peroxidase complex (ABC) method with the biotinylated tyramine-glucose oxidase amplification in this study (Kuramoto et al., 2009). Briefly, the free-floating sections were incubated in PBS containing 0.3% H_2_O_2_ for 10 min at room temperature. After washing in PBS, the sections were blocked in PBS with 0.3% Triton X-100 (PBS-T) and 1% normal donkey serum for 1 h at room temperature. This was followed by overnight incubation with the rabbit anti-mCherry antibody or the rabbit anti-EGFP antibody in PBS-T containing 1% normal donkey serum and 0.02% sodium azide (PBS-TSS) at room temperature. After washing in PBS-T, the sections were incubated with the biotinylated secondary antibody against rabbit (1:1000; Millipore, Billerica, MA, USA) in PBS-TSS for 2 h at room temperature. After washing in PBS-T, the sections were incubated with an ABC (1:200; Vectastain ABC Elite kit, Vector Laboratories, Burlingame, CA, USA) in PBS at room temperature for 1 h. After washing in 0.1 M phosphate buffer (PB; pH 7.4), the sections were incubated for 30 min in the biotinylated tyramine-glucose oxidase reaction mixture containing 1.25 μM biotinylated tyramine, 2 mg/ml of glucose oxidase (Nacalai Tesque, Kyoto, Japan; 259 U/mg), 2 mg/ml of beta-D-glucose, and 1% BSA in 0.1 M PB. After washing in PBS, the sections were again incubated for 1 h with an ABC in PBS. Finally, the bound peroxidase was developed black by staining with nickel-enhanced coloring solution (0.2 mg/ml diaminobenzidine, 0.03% H_2_O_2_, 0.03% nickel chloride in tris-buffered saline).

### Immunofluorescent Staining

For sections from FB-injected materials, we first took photomicrographs of all the FB- and GFP-double-labeled cells in the sections with a ×20 objective under an epifluorescence microscope BZ-9000 (Keyence, Osaka, Japan). Secondly, we processed the sections for immunofluorescent staining with the following antibodies: anti-calretinin (CR) rabbit serum (Swant, Bellinzona, Switzerland; 1/10,000), anti-neuronal nitric oxide synthase (nNOS) rabbit IgG (Sigma; 1/2000), anti-neuropeptide Y (NPY) rabbit serum (Sigma; 1/2000), anti-PV mouse IgG (Swant; 1/2000 from ascites), anti-SS rabbit affinity purified polyclonal antibody (Millipore; 1/300), and anti-VIP rabbit polyclonal antibody (Immunostar, Hudson, WI, USA; 1/400). The sections were incubated with one of the primary antibodies overnight at room temperature in PBS-TSS. After washing with PBS-T, the sections were further incubated with Alexa594-conjugated secondary antibody against rabbit or mouse IgG (Molecular Probes, Eugene, OR, USA) for 2 h at room temperature. The sections were mounted onto non-coated glass slides in PBS. When the FB- and GFP-double-labeled cells were negative for one of neurochemical markers, the sections were re-incubated with other neurochemical marker, and then developed with an Alexa647-conjugated secondary antibody (Molecular Probes). After immunofluorescent procedures, the sections were incubated with Hoechst33342 (Wako, Tokyo, Japan) to identify laminar structures of neocortex (Cx).

For double or triple immunofluorescent staining, some sections from the AAV injected materials were incubated with several primary antibodies in PBS-TSS (Table 1), followed by a mixture of corresponding secondary antibodies of different fluorescences without cross-reactivity for the other primary antibodies in individual combinations.

**Table 1:** **List of primary antibodies**.

Antigen	Animal	Dilution	Source
Calretinin	Rabbit	1:10000	Swant (7699/4)
GABA	Rabbit	1:1000	Sigma (A-2052)
GFP	Guinea pig	0.2 μg/ml	Dr. Takeshi Kaneko
mRFP	Guinea pig	0.2 μg/ml	Dr. Takeshi Kaneko
mCherry	Rabbit	0.2 μg/ml	Raised in our lab
Neuronal NOS	Rabbit	1:1000	Sigma (N7280)
Neuropeptide Y	Mouse	1:10000	Sigma (N9528)
Parvalbumin	Rabbit	1:2000	Swant (McAB235)
Somatostatin	Rabbit	1:300	Millipore (AB5494)
Tyrosine hydroxylase	Rabbit	1:1000	Millipore (AB152)
Vasoactive intestinal peptide	Rabbit	1:400	Immunostar (20077)

### Data Analysis

Immunofluorescence was observed under either an epifluorescence microscope BZ-9000 or an confocal laser scanning microscope FV1200 (Olympus, Tokyo, Japan) with appropriate filter sets for FB (peak excitation, 365 nm; peak emission, 420 nm), GFP (peak excitation, 488 nm; peak emission, 509 nm), Alexa594 (peak excitation, 590 nm; peak emission, 617 nm) Alexa647 (peak excitation, 651 nm; peak emission, 667 nm). In the anterograde labeling experiments, we obtained the integrated images of wide area of the sections with a ×10 objective using BZ-9000, according to the manufacturer’s instruction. We took higher magnification images of individual axons with a ×60 oil immersion objective using FV1200. In the retrograde labeling experiments, we scanned sections to find FB- and GFP-double-labeled neurons with a ×20 objective using BZ-9000. We took higher magnification images of individual neurons with a ×40 objective using FV1200. The FB fluorescence was often weakened after the immunohistochemical procedures, although some FB-labeled granules can be detected in the perikarya. When the FB-fluorescence was completely quenched, we identified FB- and GFP-double-labeled neurons by carefully comparing each GFP-labeled cell and its surrounding structures with those structures in the photomicrographs taken before immunostaining.

## Results

### Selective Labeling in Neocortical GABAergic Neurons

**Figure 1:**
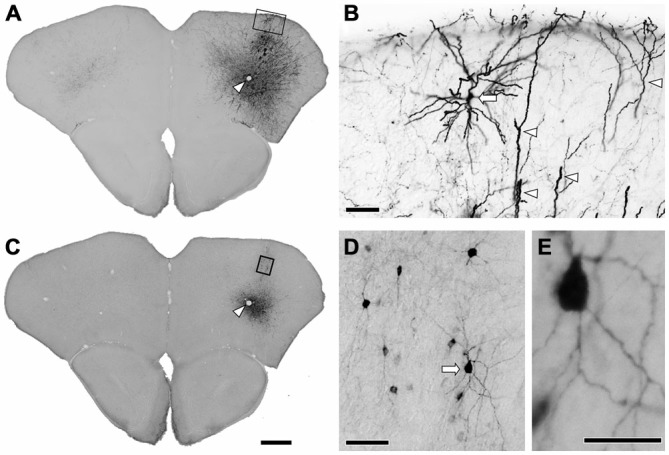
**Immunohistochemistry after the injection of viral mixture. (A)** Low magnification lightfield photomicrographs showing green fluorescent protein (GFP)-immunoreactive cells after the infection of adeno-associated viruse (AAV)-CAG-GFP. **(B)** Higher magnification from the rectangle in **(A)**. Apical dendrites (arrowheads) and the pyramidal cell (arrow) were strongly visualized by GFP-immunohistochemistry. **(C)** Low magnification lightfield photomicrographs of the adjacent section showing mCherry-immunoreactive cells by the infection of AAV-Ef1a-DIO-hChR2-mCherry. Arrowheads in **(A,C)** indicate the same vessels. **(D)** Higher magnification from the rectangle in **(C)**. Nonpyramidal neurons were visualized by mCherry-immunohistochemistry. Note that any features of pyramidal neurons were not identified among the mCherry-immunoreactive structures. **(E)** Higher magnification from the arrow in **(D)**. Monomeric cherry-immunoreactive neurons have smooth dendrites. Scale bars = 400 μm in **(A)** (for **C**); 50 μm in **(B,D)**; 20 μm in **(E)**.

**Figure 2:**
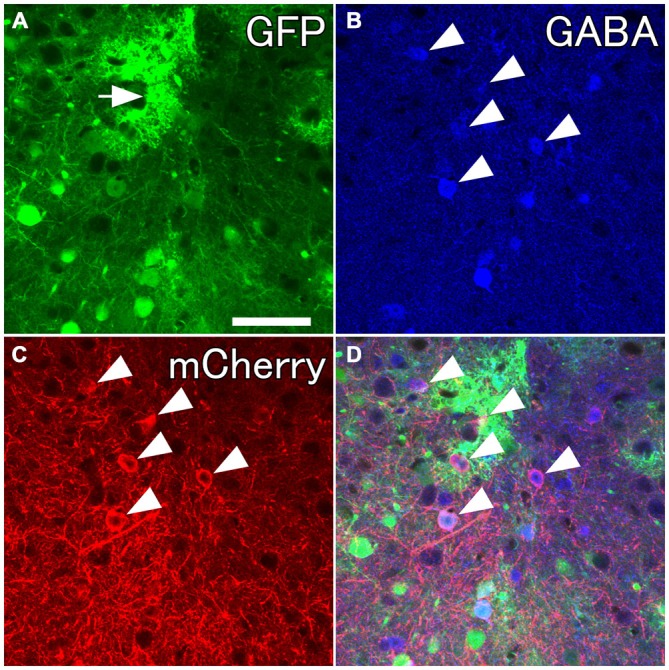
**Selective labelings of neocortical GABAergic neurons.** Fluorescent photomicrographs showing GABA-immunoreactivity in mCherry-immunoreactive neurons. **(A)** GFP-immunoreactive neurons and glia-like cells (the arrow) around the injection site. **(B–D)** Almost all mCherry-immunoreactive neurons are co-localized with GABA-immunoreactivity. Scale bar = 50 μm.

Several previous reports have demonstrated highly specific expression of Cre recombinase in GAD67-Cre knock-in mice (Higo et al., 2009; Wu et al., 2011; Saito et al., 2013). However, no report has examined specific expression of a reporter gene in neocortical GABAergic neurons using an AAV vector. In order to ensure selective expression of the reporter gene using the AAV vector, we first injected the viral mixture (AAV-Ef1a-DIO-hChR2-mCherry and AAV-CAG-GFP) into the frontal cortex of GAD67-Cre knock-in mice. The diameters of core injections were estimated as 0.4–0.6 mm. The injection sites were confined in frontal cortex, mainly orbitofrontal cortex (Figure 1). Immunohistochemistry for GFP demonstrated the structures of pyramidal cells such as thick apical dendrites and spiny dendrites, and glia cells (Figure 1B). In contrast, immunohistochemistry for mCherry in the adjacent section demonstrated only nonpyramidal cells with smooth dendrites (Figure 1D). Any pyramidal structures like apical dendrites were not observed in the mCherry-immunoreacted sections. To further confirm whether mCherry-immunoreactive neurons are only GABAergic neurons, we examined the co-localization of GABA-immunoreactivity in mCherry-immunoreactive neurons. Almost all mCherry-immunoreactive neurons (97%) exhibited GABA-immunoreactivity (Figure 2), whereas GFP-immunoreactivity were found mainly in GABA-negative neurons and occasionally in glia cells. Together, we concluded that the injection of AAV-Ef1a-DIO-hChR2-mCherry into the Cx of GAD67-Cre knock-in mice can induce the expression of hChR2-mCherry in GABAergic neurons, but not glutamatergic neurons.

### Anterograde Labeling of Neocortical GABAergic Neurons

**Figure 3:**
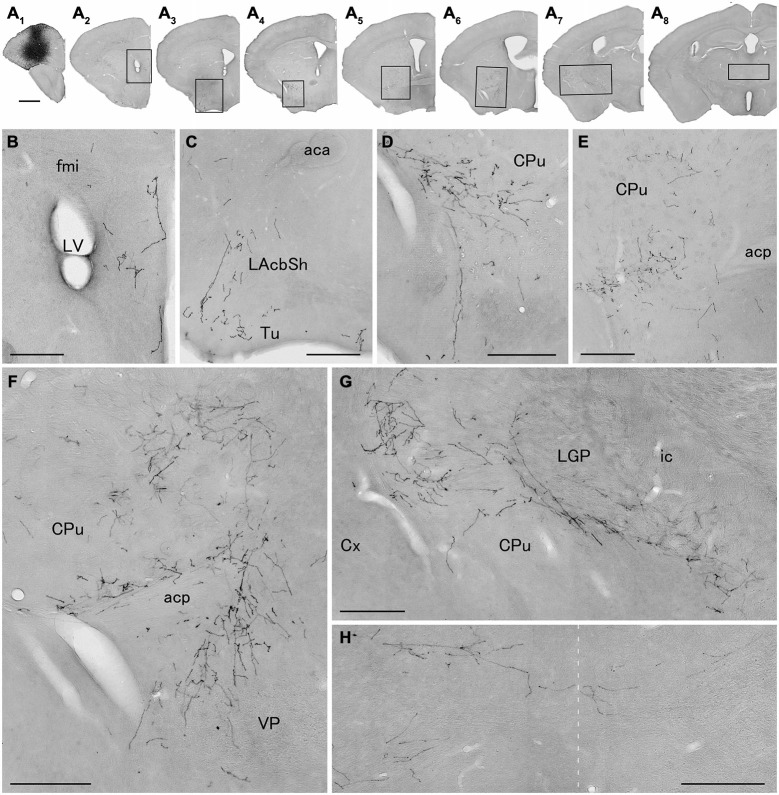
**Distribution of axonal fibers form neocortical GABAergic projection neurons in case mRT217.** Low **(A)** and high **(B–H)** magnification lightfield photomicrographs showing mCherry-immunoreactive fibers in selected sections after the injection of AAV-Ef1a-DIO-hChR2-mCherry mainly in the orbital cortex **(A1)**. The mCherry-immunoreactive fibers were found in the dorsal peduncular and infralimbic cortex **(A2,B)**, in the lateral accumbens shell and olfactory tubercle **(A3,C)**, in the ventral caudate putamen **(A4–A7,D–G)**, in the ventral pallidam **(A6,F)**, in the lateral globus pallidus **(A7,G)**, and in the mediodorsal thalamic nucleus **(A8,H)**. Note that mCherry-immunoreactive fibers were also found in the contralateral mediodorsal thalamic nucleus. The dashed line indicates the midline of the brain. aca, anterior commissure, anterior part; acp, anterior commissure, posterior part; CPu, caudate putamen; Cx, neocortex; fmi, forceps minor of the corpus callosum; ic, internal capsule; LAchSh, lateral accumbens shell; LGP, lateral globus pallidus; LV, lateral ventricle; Tu, olfactory tubercle; VP, ventral pallidum. Scale bars = 1 mm in **(A)**; 200 μm in **(B–H)**.

**Figure 4:**
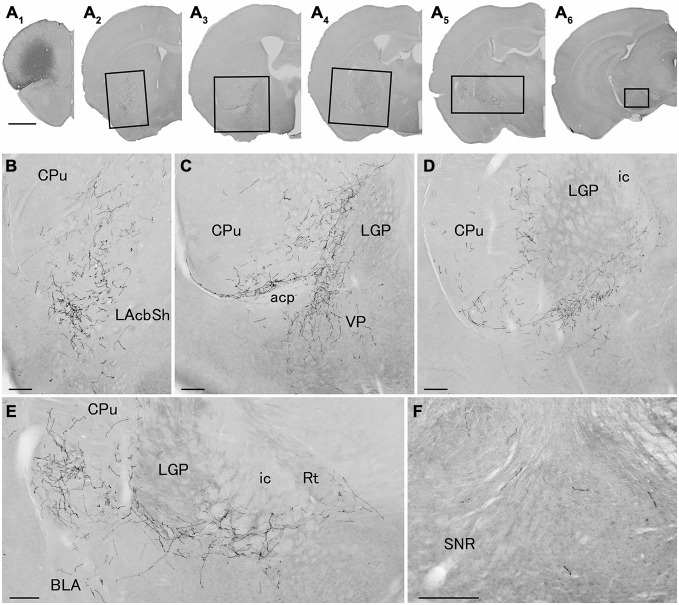
**Distribution of axonal fibers form neocortical GABAergic projection neurons in case mRT218.** Low **(A)** and high **(B–F)** magnification lightfield photomicrographs showing mCherry-immunoreactive fibers in selected sections after the injection of AAV-Ef1a-DIO-hChR2-mCherry mainly in the orbital cortex **(A1)**. The mCherry-immunoreactive fibers were found in the lateral accumbens shell **(A2,B)**, in the ventral CPu **(A2–A5,B–E)**, in the ventral pallidam **(A3,C)**, in the LGP **(A3–A5,C–E)**, in the basolateral amygdala **(A5,E)**, and in the substantia nigra **(A6,F)**. Note that most of mCherry-immunoreactive fibers appear to travel below the LGP, although some beaded axonal fibers were also found in the ventral part of LGP. BLA, basolateral amygdaloid nucleus, anterior part; Rt, reticular thalamic nucleus; SNR, substantia nigra pars reticulata. Scale bars = 1 mm in **(A)**; 200 μm in **(B–F)**.

**Figure 5:**
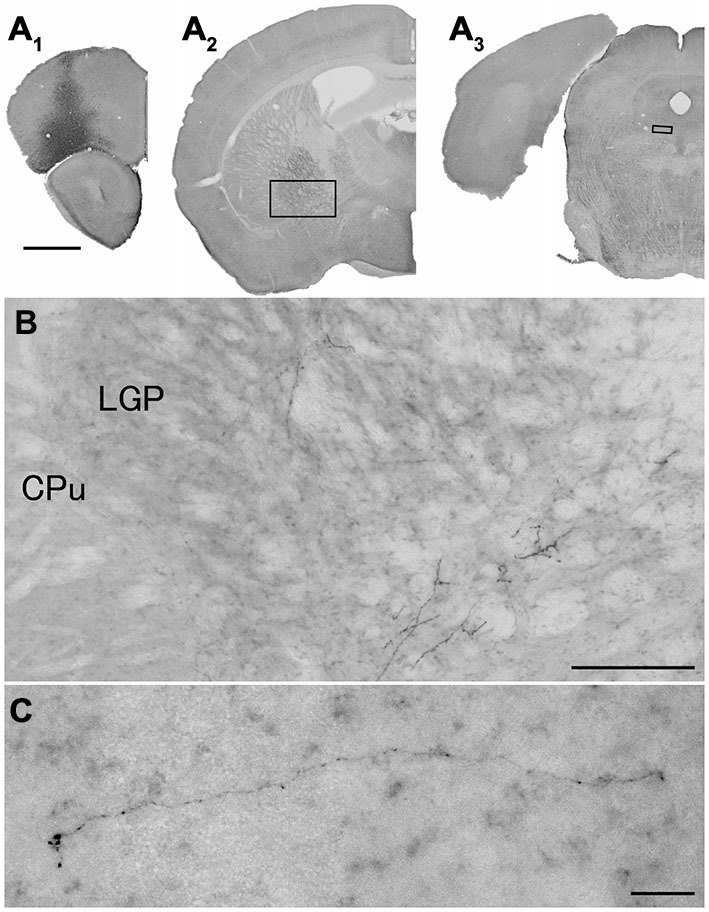
**Distribution of axonal fibers form neocortical GABAergic projection neurons in case mRT210.** Low **(A)** and high **(B,C)** magnification lightfield photomicrographs showing mCherry-immunoreactive fibers in selected sections after the injection of AAV-Ef1a-DIO-hChR2-mCherry mainly in the orbital cortex **(A1)**. The mCherry-immunoreactive fibers were found in the LGP **(A2,B)** and in the dorsal raphe nucleus **(A3,C)**. Scale bars = 1 mm in **(A)**; 200 μm in **(B)**; 30 μm in **(C)**.

**Figure 6:**
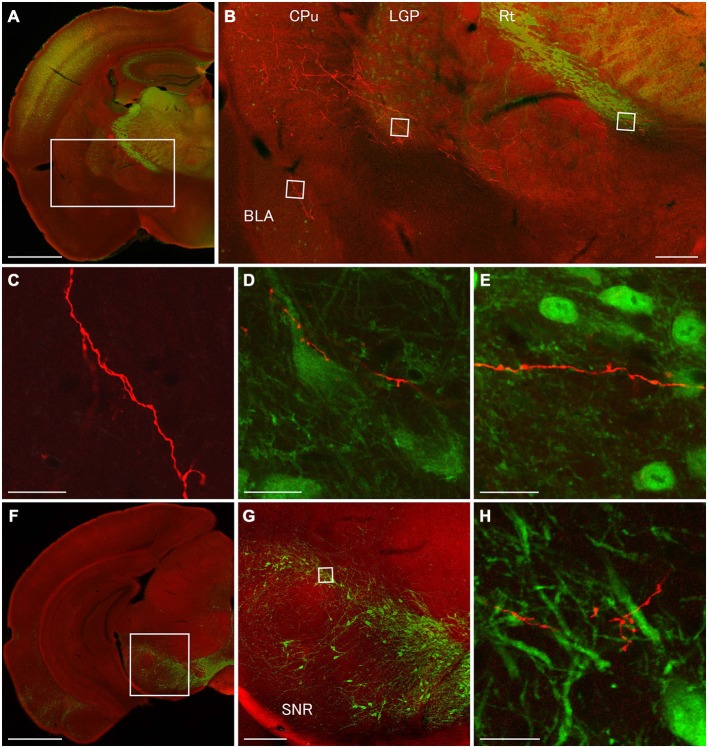
**Terminal specializations of corticofugal GABAergic projection.** Low **(A,F)** and high **(B–E,G,H)** magnification confocal laser scanning images showing mCherry-immunoreactive fibers (red) after the injection of AAV-Ef1a-DIO-hChR2-mCherry mainly in the orbital cortex. **(A–E)**: parvalbumin (PV)-immunoreactivity (green) clearly shows the boundaries for the CPu, LGP, and Rt. Monomeric Cherry-immunoreactive fibers were found in the CPu **(B)**, basolateral amygdala **(B,C)**, LGP **(B,D)**, and Rt **(B,E)**. The terminal specialization are mainly beaded **(C–E)** and very ocassionally stalked **(D,E)**. Note that mCherry-immunoreactive fibers established multiple synaptic appositions with the somata and dendrite of the PV-immunoreactive neuron in the LGP **(D)**. **(F–G)**: Tyrosine hydroxylase (TH)-immunoreactivity (green) clearly shows the distribution of dopaminergic neurons in the substantia nigra pars compacta. The terminal specialization are mainly beaded in the substantia nigra pars compacta **(G)**. Scale bars = 1 mm in **(A,F)**; 200 μm in **(B,G)**; 20 μm in **(C–E,H)**.

We next explored the possibility that axonal fibers from neocortical GABAergic projection neurons may innervate into several brain regions. In order to visualize axonal fibers of GABAergic neurons, we injected 50 nl of AAV-Ef1a-DIO-hChR2-mCherry into the frontal cortex of GAD67-Cre knock-in mice (*n* = 18). We confirmed the injection sites were relatively larger (0.6–1.0 mm) and were confined only in frontal cortex (Figures 3–5) in 10 mice. These 10 mice were chosen for further analysis. Neurons labeled by AAV-Ef1a-DIO-hChR2-mCherry were found within the injection sites such as the dorsolateral, lateral, and ventral orbital, and primary and secondary motor cortices. In addition, mCherry-immunoreactive fibers were found throughout the rostral-caudal extent of the brain. The numbers and intensities of mCherry-immunoreactive fibers were diverse across animals, presumably because of the different sizes of injection sites. In contrast, the patterns of mCherry-immunoreactive fibers were similar across all animals injected, with some differences (Table 2). Abundant mCherry-immunoreactive fibers with en passant synapses were observed in the cerebral cortex, basal ganglia and thalamus (Figure 6). Additionally mCherry-immunoreactive fibers appeared in the amygdala (Figure 6C) in three cases, and in the dorsal raphe nucleus in one case (Figure 5C). The details of axonal distribution are briefly summarized below.

**Table 2:** **Relative density of GABAergic fibers in various brain structures from representative injections**.

Brain structure	199	200	209	210	217	218	219	222
Dorsal peduncular cortex	++			++	++	+	++	+
Medial orbital cortex	++		+		++	++	++	++
Agranular insular cortex	+	+	+	++	+	+	+	+
Granular/Dysgranular insular cortex					++	+
Cingulate cortex				+	+	+
Piriform cortex	+		+		++	++
Claustram	++		+	+	++	++	++	+
Motor cortex				++	+	++	+	+
Somatosensory cortex				+	++	++	++
**Basal ganglia**
Caudate putamen	++	++	++	++	++	+++	+++	+++
Nucleus accumbens core			+		++	++
Nucleus accumbens shell	+	+	+	++	++	++	++	+++
Olfactory tubercle	+	+	+	++	+++	++	+	+
Ventral pallidum	++	+	+	++	+++	+++	++	+
Lateral globus pallidus	+	+	++	++			+	++
Medial globus pallidus					+	+	++	++
Substantial nigra					+	+	+
**Septum**
Medial septum		+			+		+
Diagonal band of broca		+			+		++
**Thalamus/Epithalamus**
Mediodorsal thalamic nucleus	+			+	+		+
Centromedial thalamic nucleus		+			+
Lateral habenular nucleus	+
**Amygdala**
Medial amygdaloid nucleus					+	+
Anterior amygdaloid area						+	+
Basolateral amygdala						+
**Parahippocampal region**
Presubiculum		+
Lateral entorhinal cortex						+
Perirhinal cortex						+
**Pons**
Dorsal raphe nucleus				+

In the cerebral cortex, mCherry-immunoreactive fibers were frequently found in the ipsilateral hemisphere: the insular, dorsal peduncular, motor, and somatosensory cortices (Table 2). In two cases (mRT200, 218), the mCherry-immunoreactive fibers were observed in the parahippocampal regions: presubiculum, perirhinal and lateral entorhinal cortices. In some cases, the mCherry-immunoreactive fibers also occurred in the contralateral hemisphere: the agranular insular, infralimbic, and orbitofrontal cortices. These axonal fibers were distributed mainly in layers 1, 2, and 6.

The basal ganglia was the most densely mCherry-immunoreactive structure across all animals injected (Figures 3–5; Table 2). Most of labeled fibers formed en passant synapses in the anterior part of CPu, but not axonal bundles as passing fibers. Farther caudally, labeled fibers were concentrated in the ventral part of both CPu and LGP (Figures 3D–G, 4C–E, 5B). In addition, labeled fibers were frequently observed in the medial globus pallidus, accumbens core and shell, olfactory tubercle (Tu), substantia innominata, and ventral pallidum (VP; Figures 3, 4 and Table 2), and rarely in the contralateral CPu and VP (data not shown). It is worth noting that mCherry-immunoreactive fibers were found in the substantia nigra in three cases (Figure 4F and Table 2).

In the thalamus, mCherry-immunoreactive fibers were found occasionally in mediodorsal thalamic nucleus in the four cases, and very rarely in the contralateral mediodorsal thalamic nucleus in the case mRT217 (Figure 3H). In two cases (mRT217, 218), the mCherry-immunoreactive fibers formed en passant synapses in the reticular thalamic nucleus (Rt; Figure 4E).

In order to discover postsynaptic elements of GABAergic projection neurons, we carried out double fluorescent stainings with anti-PV and anti-mCherry antibodies for the nuclei of basal ganglia and reticular thalamus (Hontanilla et al., 1998; Kita and Kita, 2001; Miyamoto and Fukuda, 2015), and with anti-tyrosine hydroxylase (TH) and anti-mCherry antibodies for the substantia nigra pars compacta. We reconfirmed numerous en passant synapses on mCherry-immunoreactive fibers in the LGP where strong PV-immunoreactivity appeared (Figures 3G, 4D,E, 6B,D). Moreover, we found multiple synaptic appositions of mCherry-immunoreactive fibers on the somata and dendrite of the PV-immunoreactive neuron (Figure 6D). To our knowledge, this is the first study suggesting that neocortical neurons may innervate neurons in the LGP (Gerfen, 2004). Although en passant synapses on mCherry-immunoreactive fibers were confirmed in both the Rt and substantia nigra pars compacta, we could not find any candidates of synaptic appositions on either PV- or TH- immunoreactive neurons (Figures 6E,H). Further observations with an electron microscope will be required to discover postsynaptic elements for those en passant synapses.

### Neurochemical Markers of Corticofugal GABAergic Neurons

We have demonstrated the distribution of axonal fibers from neocortical GABAergic projection neurons. Since neocortical GABAergic neurons are classified into subpopulations by electrophysiological, morphological, and neurochemical properties (Gonchar and Burkhalter, 1997; Markram et al., 2004; Ascoli et al., 2008; Kubota et al., 2011; Sohn et al., 2014; Zeisel et al., 2015), we next examine which GABAergic subpopulations project to each brain region. To identify corticofugal GABAergic neurons, we injected the retrograde tracer FB into several brain regions: the ventral part of CPu, mediodorsal thalamic nucleus, or dorsal raphe nucleus.

**Figure 7:**
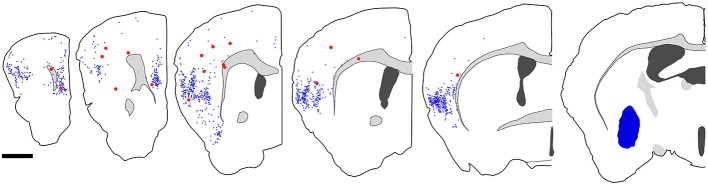
**Distribution of fast blue (FB)-labeled and FB- and GFP-double-labeled neurons in the cerebral cortex after FB injection into the ventral part of CPu.** Five coronal section outlines illustrating the distribution of retrogradely labeled neurons in the Cx after FB injection into the ventral part of CPu and VP. One coronal section outline (in the rightmost) showing only the injection site. FB-labeled neurons (blue dots) were found mainly in the insular cortex, and secondary in the medial prefrontal cortex. FB- and GFP-double-labeled neurons (red dots) were found mainly in the anterior part of frontal cortex. Note that the occurrence of FB-and GFP-double-labeled neurons is not correlated with the density of FB-labeled neurons. Scale bar = 1 mm.

**Figure 8:**
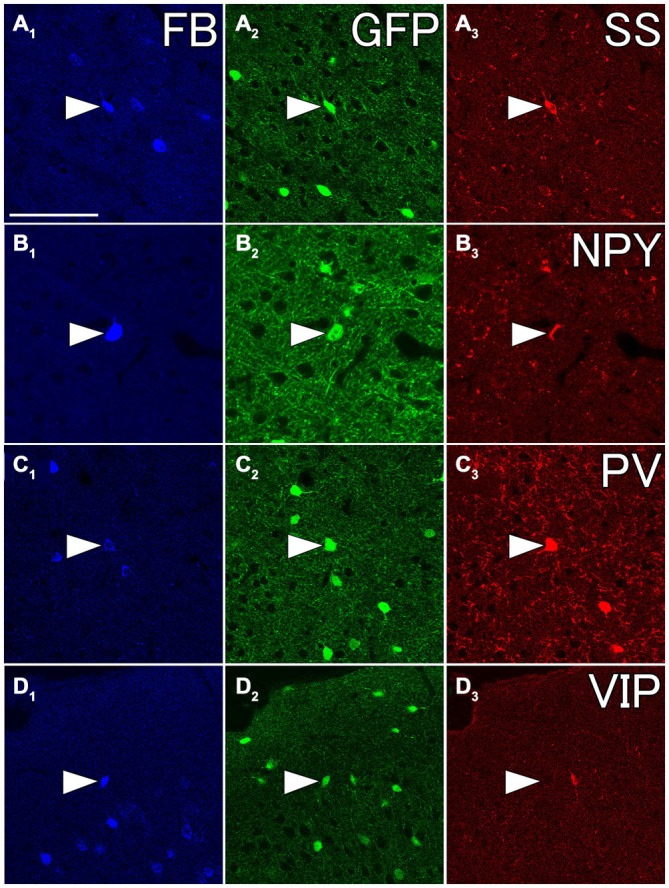
**Fluorescent photomicrographs showing neurochemical characterization of FB- and GFP-double labeled neurons in the Cx.** Most of FB- and GFP-double labeled neuron were co-labeled with somatostatin (SS) **(A1–A3)** and neuropeptide Y (NPY) **(B1–B3)**, and some of them were with PV **(C1–C3)**, but not with vasoactive intestinal peptide (VIP) **(D1–D3)**. The arrowheads in each row indicate the same triple- or double labeled neurons in **(A–D)**. Scale bar = 100 μm in **(A)** (for **B–D**).

We injected 100 nl of FB into the ventral part of CPu of GAD67-GFP knock-in mice (*n* = 8). We confirmed the injection sites were mainly confined in the ventral part of CPu and LGP. Injections of FB into the ventral part of CPu resulted in numerous FB-labeled neurons mainly in layers 5 and 2/3 of the frontal cortex, consistent with the previous report in Gabbott et al. (2005), and very occasionally FB- and GFP-double labeled neurons in the frontal cortex (Figure 7). Interestingly, FB- and GFP-double-labeled neurons did not mingle with numerous FB-labeled neurons. Rather FB- and GFP-double-labeled neurons were found in the regions with sparsely FB-labeled neurons. The FB- and GFP-double labeled neurons were observed mainly in infragranular layers, but also in layer 2/3 and the subcortical white matter. Next, we further examined the neurochemical properties of FB- and GFP-double labeled neurons using antibodies for CR, nNOS, NPY, PV, SS, and VIP. Triple labeling demonstrated that most of FB- and GFP-double-labeled neurons exhibited SS-immunoreactivity (53%: 41/78) or NPY-immunoreactivity (43%: 6/14). Some of them exhibited PV-immunoreactivity (7%: 4/61), and none exhibited CR- (0%: 0/14), nNOS- (0%: 0/24), or VIP- (0%: 0/40) immunoreactivity (Figure 8).

**Figure 9:**
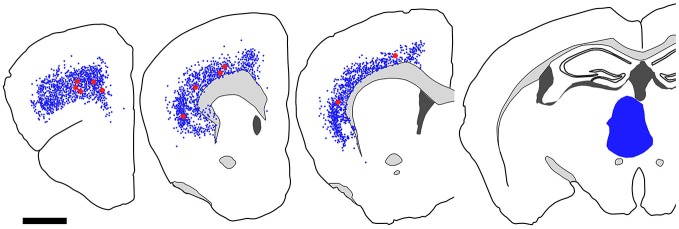
**Distribution of FB-labeled and FB- and GFP-double-labeled cells in the cerebral cortex after FB injection into the mediodorsal thalamic nucleus.** Four coronal section outlines illustrating the distribution of retrogradely labeled neurons in the Cx after FB injection into the mediodorsal thalamic nucleus. One coronal section outline (in the rightmost) showing only the injection site. FB-labeled neurons (blue dots) were found mainly in layer 6 of Cx. FB- and GFP-double-labeled neurons (red dots) were also found in layer 6. Scale bar = 1 mm.

We injected 100 nl of FB into the mediodorsal thalamic nucleus of GAD67-GFP knock-in mice (*n* = 13). We confirmed the injection sites were mainly confined in the mediodorsal thalamic nucleus (*n* = 8). We chose three brain samples for further analysis after scanning sections under an epifluorescence microscope, because we could find few FB- and GFP-double labeled neurons in most samples. Injection of FB into the mediodorsal thalamic nucleus resulted in numerous FB-labeled neurons in layer 6 of the frontal cortex, consistent with the previous report in Gabbott et al. (2005), and very occasionally FB- and GFP-double labeled neurons in layer 6 (Figure 9). Although we further examined the neurochemical properties of FB- and GFP-double labeled neurons, we failed to identify any neurochemical properties of FB- and GFP-double labeled neurons (0/24 for CR; 0/21 for PV; 0/23 for SS; 0/17 for VIP).

We injected 100 nl of FB into the dorsal raphe nucleus of GAD67-GFP knock-in mice (*n* = 7). We confirmed the injection sites were mainly in the dorsal raphe nucleus and superior colliculus. Injection of FB resulted in numerous FB-labeled neurons in upper layer 5 and lower layer 3 of the frontal cortex, and occasionally in layer 2 and 6, consistent with the previous report in Gabbott et al. (2005). Although we made FB-injection into the dorsal raphe nucleus of 7 GAD67-GFP knock-in mice and scanned five frontal sections from each brain sample, we found only 7 FB- and GFP-double labeled neurons. Since the number of sections with FB- and GFP-double labeled neurons was limited, we further tested one neurochemical property using the antibody for SS. Only one FB- and GFP-double labeled neuron exhibited SS-immunoreactivity (data not shown).

### Distribution of Axonal Fibers from both GABAergic and Glutamatergic Neurons

We have confirmed that subpopulations of GABAergic neurons in the frontal cortex project to ventral part of CPu. Since glutamatergic neurons in the frontal cortex also project to the subcortical regions, it is absolutely essential to understand whether both GABAergic and glutamatergic neurons innervate the same brain regions. We next examined the axonal trajectories from both neocortical GABAergic and glutamatergic neurons. In order to visualize axonal fibers of GABAergic and glutamatergic neurons, we injected a mixture of AAV-Ef1a-DIO-hChR2-mCherry and AAV-CAG-GFP into the frontal cortex of GAD67-Cre knock-in mice.

**Figure 10:**
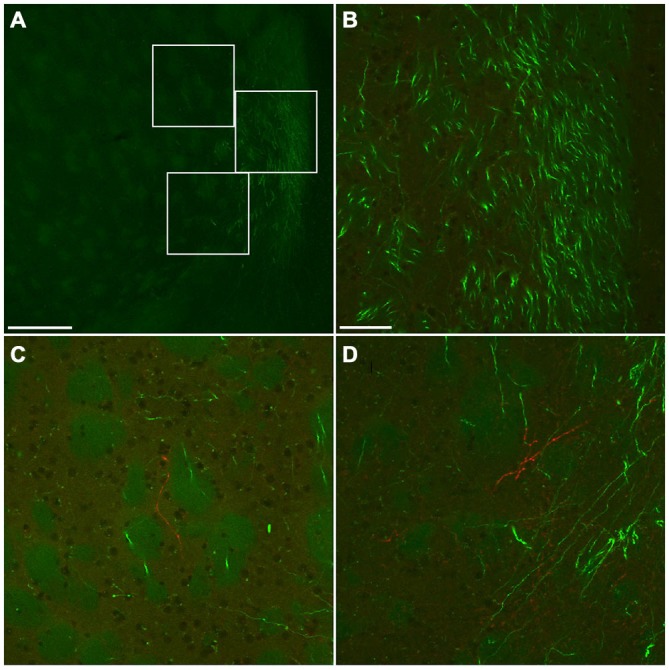
**Fluorescent photomicrographs showing the distributions of GFP- or mCherry-immunoreactive fibers in the ventromedial part of CPu.** Low **(A)** and high **(B–D)** magnification confocal laser scanning images showing mCherry- or GFP-immunoreactive fibers after the injection of AAV-Ef1a-DIO-hChR2-mCherry and AAV-CAG-GFP into the frontal cortex. No mCherry-immunoreactive fiber was found in the main trajectory of GFP-immunoreactive fibers **(B)**. In contrast, some mCherry-immunoreactive fibers were found in the marginal zone of main trajectory of GFP-immunoreactive fibers **(C,D)**. The upper, middle, bottom rectangles in **(A)** correspond with **(C)**, **(B)**, and **(D)**, respectively. Scale bars = 250 μm in **(A)**; 50 μm in **(B)** (for **C,D**).

Numerous GFP-immunoreactive fibers mainly from neocortical glutamatergic neurons were found throughout the rostral-caudal extent of CPu. A few mCherry-immunoreactive fibers from GABAergic neurons were found in the anterior CPu, whereas many were found in the posterior CPu (data not shown). Interestingly, mCherry-immunoreactive fibers were found mainly in the brain regions where a few GFP-immunoreactive fibers were found. For example, at the level of the crossing of the anterior commissure, abundant bundles of GFP-immunoreactive fibers were found in the ventromedial CPu, while mCherry-immunoreactive fibers were found mainly in further lateral part of main GFP-immunoreactive area (Figure 10). Together, GABAergic axonal fibers from the frontal cortex may innervate into different brain areas from glutamatergic axonal fibers.

## Discussion

We report that a subset of GABAergic neurons in the mouse frontal cortex project widely to cortical areas and subcortical nuclei throughout the forebrain and midbrain. They project primarily to the basal ganglia including the CPu, VP, LGP, nucleus accumbens, and Tu (Table 2). In some cases, they also project to the mediodorsal thalamic nucleus, diagonal band of Broca, medial globus pallidus, substantia nigra, and dorsal raphe nucleus. In addition, retrograde tracing experiments also confirm neocortical GABAergic projections to basal ganglia, thalamic nuclei, and dorsal raphe nucleus. The majority of corticostriatal GABAergic projection neurons are SS-positive neurons and some are PV-positive neurons, while the others are unidentified by representative neurochemical markers. In addition, corticothalamic GABAergic projection neurons are also unidentified by representative neurochemical markers. Together those results indicate that corticofugal GABAergic projection neurons from the frontal cortex would consist of several distinct subpopulations of GABAergic neurons.

### Technical Considerations

Although Cre-dependent expression of hChR2-mCherry in GAD67-Cre knock-in mice labeled nonpyramidal neurons, a natural concern is whether the apparent labeling could be specific. We think this concern is unlikely for several reasons. First, mCherry-immunoreactivity by Cre-dependent excision were found in nonpyramidal cells, but not in pyramidal and glia cells. In addition, we confirmed almost all mCherry-immunoreactive cells were positive for GABA-immunoreactivity. Second, retrograde tracing experiments were strongly consistent with the anterograde labeling by Cre-dependent excision. Third, our results are partly consistent with the previous report in Lee et al. (2014), which has described corticofugal GABAergic projection neurons in the medial frontal cortex. Another potential concern is the sensitivity of our methods to detect axonal fibers. We took three methodological improvements to deal with this technical difficulty. First, we chose hChR2-mCherry as a reporter gene, because it binds the plasma membrane, and may therefore diffuses faster than cytosolic proteins. Second, relatively longer survival period after the virus injection allows hChR2-mCherry to diffuse into axonal fibers even in remote brain areas. Third, biotinylated tyramine-glucose oxidase amplification enhances the mCherry-immunoreactivity. In our pilot study, we found more enhanced signals and more axonal fibers by biotinylated tyramine-glucose oxidase amplification than by only conventional ABC method. Indeed, mCherry-immunoreactive fibers were found in the remote brain area such as the dorsal raphe nucleus (Figure 5C). Therefore, our experimental procedures makes it possible to detect the reporter gene of the AAVs even in the axonal projection fibers of remote brain areas.

The volume of viral injection is the primary factor to obtain strong labeling, especially when the distribution of axonal fibers is analyzed. In this study, the maximal volume of viral injection is limited to less than 50 nl, which size is roughly equal with a 350 μm cube. Considering that an injection solution is infused into extracellular space, a diameter of injection would be much larger than 350 μm. Indeed, the diameters of injection sites are 400–1000 μm in this study, although we carefully injected a small volume of viral solution several times for over 30 min in order to deal with this issue. This technical limitation by a pressure injection could be improved by a more sophisticated method iontophoresis (Wang et al., 2014).

In the present study, we demonstrated corticofugal GABAergic projection neurons qualitatively, since the labeling efficiency of the viral-vector-mediated anterograde tracer were variably across animals. This technical limitation would be general among neuroanatomical tracers. Indeed we carefully injected the small volume of isosmotic viral solution into the frontal cortex to avoid any alternation such as tissue damage, but it was hard to control quality of injection. Recently single cell labeling techniques (Pinault, 1996; Matsuda et al., 2009) have succeeded to provide quantitative data like numbers of synaptic bouton. In near future, quantitative analysis with single cell labeling techniques will reveal more detailed evidence about the neural network of corticofugal GABAergic projection neurons.

### Previous Studies

Several studies have demonstrated glutamatergic projection fibers from the rodent frontal cortex to the nuclei throughout the forebrain and brainstem. In this study, the distribution of projection fibers, which are non-selective GFP-labeled (Figure 10), are mainly consistent with previous reports in Morino et al. (1994), Zaborszky et al. (1997), Hoover and Vertes (2011) and Kim and Lee (2012). For example, abundant GFP-labeled fibers from the frontal cortices were found in the CPu, VP, mediodorsal thalamic nucleus, and dorsal raphe nucleus, and occasionally in the cerebral peduncle. Together, the vast majority of GFP-labeled fibers in the subcortical areas would originate from neocortical glutamatergic neurons.

Our experiments confirm and extend the recent work describing GABAergic projection neurons in the medial prefrontal cortex using Dlxi12b–Cre transgenic mice (Lee et al., 2014). They injected relatively larger volume of Cre-dependent AAVs into the medial prefrontal cortex of Dlxi12b–Cre transgenic mice, and demonstrated GABAergic projection fibers in the dorsal striatum, nucleus accumbens, claustrum, and basolateral amygdala. In this study, we also found GABAergic projection fibers in more various brain regions: mainly in ventral part of CPu, nucleus accumbens, claustrum, Tu, VP, LGP, mediodorsal thalamic nucleus and substantia nigra (Table 2). This is presumably because our method to visualize the reporter gene may be more sensitive.

In order to examine the neurochemical properties of GABAergic projection neurons in the prefrontal cortex, Lee et al. (2014) injected CTB-Alexa488 into the nucleus accumbens of either PV-IRES-Cre, VIP-IRES-Cre, or SS-IRES-Cre crossed to TdTomato reporter line (Ai14). They found retrogradely labeled neurons in 6 of 144, 5 of 100, and 0 of 156 TdTomato-expressing neurons within the medial prefrontal cortex of PV-IRES-Cre × Ai14, VIP-IRES-Cre × Ai14, and SS-IRES-Cre × Ai14 mice, respectively. Considering that PV-, VIP-, and SS-expressing subpopulations would constitute the vast majority of GABAergic neurons in the Cx (Uematsu et al., 2008; Kubota et al., 2011; Sohn et al., 2014), their results indicate most of GABAergic projection neurons exhibit either PV- or VIP-immunoreactivity, but not SS-immunoreactivity. In contrast, we found majority of GABAergic projection neurons from the frontal cortex to the ventral part of CPu are SS-immunoreactive neurons, very occasionally PV-immunoreactive neurons, but not VIP-immunoreactive neurons. This discrepancy could be due to several methodological differences between two studies: first, different retrograde tracers were used. Second, the retrograde tracers were injected into different brain regions. Third, the different methods to detect each GABAergic subpopulation were employed. Indeed we employed GAD67-GFP knock-in mice to detect GABAergic neurons and identify GABAergic subpopulations using antibodies against neurochemical markers. In contrast, Lee et al. (2014) employed three different knock-in mice to identify GABAergic subpopulations. In this study, we first detected the general population of retrogradely labeled GABAergic neurons, and then examined their neurochemical properties. Therefore, this approach allows us to conclude which subpopulation constitutes GABAergic projection neurons. In our conclusion, the majority of GABAergic projection neurons in the mouse frontal cortex project to the subcortical areas such as ventral part of CPu and LGP, and express SS. We also note that a further systematic study needs to reveal this discrepancy about neurochemical properties of corticofugal GABAergic projection neurons.

### Circuitry and Functional Significance

Several studies have demonstrated corticocortical GABAergic projection neurons in rodents, carnivores, and monkeys (Peters et al., 1990; McDonald and Burkhalter, 1993; Tomioka et al., 2005; Tomioka and Rockland, 2007; Higo et al., 2007, 2009). There is increasing evidence that corticocortical GABAergic projection neurons are relatively homogeneous subpopulation of GABAergic neurons containing SS (Tomioka et al., 2005; Tomioka and Rockland, 2007; Higo et al., 2007, 2009; Jinno et al., 2007; McDonald et al., 2012; Melzer et al., 2012; Caputi et al., 2013; McDonald and Zaric, 2015a,b) with some exceptions of GABAergic connections (Miyashita and Rockland, 2007; Melzer et al., 2012). Especially, we have demonstrated corticocortical GABAergic projection neurons are highly homogeneous and are classified as the nNOS-, NPY-, and SS-expressing subpopulation (Tomioka et al., 2005; Tomioka and Rockland, 2007). Here, we demonstrate the majority of corticostriatal GABAergic projection neurons are classified as the SS-positive and nNOS-negative GABAergic subpopulation, indicating corticostriatal GABAergic projection neurons may be distinct from corticocortical GABAergic neurons. Considering that corticofugal GABAergic projection neurons has been reported as a subset of PV- or VIP-expressing neurons (Jinno and Kosaka, 2004; Lee et al., 2014) and we often failed to identify the neurochemical property of GABAergic projection neurons, further experiments will be required to reveal some degree of heterogeneity of GABAergic projection neurons. Together, corticofugal GABAergic projection neurons may be distinct from corticocortical projection neurons, and more heterogeneous from the neurochemical point of view.

Corticocortical GABAergic projection neurons may account for 0.5% of the neocortical GABAergic neurons (Tamamaki and Tomioka, 2010). This speculation is derived from the neurochemical identity of corticocortical GABAergic projection neurons. In this study, we could not find a specific neurochemical marker for corticofugal GABAergic projection neurons, so that we could not estimate how percentages of neocortical GABAergic neurons project corticofugally. Instead, we can trace both corticocortical and corticofugal GABAergic projection fibers in the anterograde labeling studies. We found the number of corticofugal GABAergic projection fibers are larger than that of corticocortical ones across almost all animals injected (Table 2). This result can not lead to the simple conclusion that corticofugal GABAergic projection neurons is a larger subpopulation, because we do not know how dense axonal arborizations originate from each GABAergic projection neurons. Rather, we conclude that corticofugal GABAergic projection neurons in the frontal cortex form much more synapses than corticocortical ones.

One puzzling observation is that the highly topographical overlap between corticothalamic GABAergic and glutamatergic projection fibers was demonstrated in retrograde labeling experiments (Figure 8), whereas the partially topographical overlap in the spatial territories between corticostriatal GABAergic and glutamatergic projection neurons was shown by both anterograde and retrograde labeling experiments (Figures 7, 10). This partially topographical overlap between GABAergic and glutamatergic projection neurons has been also observed among corticocortical connections (Tomioka et al., 2005). Considering that both corticostriatal and corticocortical GABAergic projection neurons are subpopulations of somatostatinergic neurons in the Cx, SS-expressing GABAergic neurons may be embedded in the unique neuronal network. Indeed, recent reports have demonstrated that SS-expressing GABAergic neurons show distinct properties among other GABAergic neurons in the local circuit (Adesnik et al., 2012; Gentet et al., 2012; Lee et al., 2012; Pfeffer et al., 2013; Xu et al., 2013; Makino and Komiyama, 2015; Pinto and Dan, 2015). Here, we demonstrated corticofugal GABAergic projections, but we did not describe the neuronal networks where corticofugal GABAergic projection neurons are embedded. Further experiments to discover their neuronal networks will reveal the different roles between corticofugal GABAergic and glutamatergic projection neurons.

## Conflict of Interest Statement

The authors declare that the research was conducted in the absence of any commercial or financial relationships that could be construed as a potential conflict of interest.
